# Computational NMR Spectroscopy of Ionic Liquids: [C_4_C_1_im]Cl/Water Mixtures

**DOI:** 10.3390/molecules25092085

**Published:** 2020-04-29

**Authors:** Giacomo Saielli

**Affiliations:** 1 CNR Institute on Membrane Technology, Unit of Padova, Via Marzolo 1, 35131 Padova, Italy; giacomo.saielli@cnr.it; 2 Department of Chemical Sciences, University of Padova, Via Marzolo 1, 35131 Padova, Italy; giacomo.saielli@unipd.it

**Keywords:** ionic liquids, mixtures, NMR, molecular dynamics simulations, density functional theory

## Abstract

In this work, I have analyzed the structure of binary mixtures of 1-butyl-3-methylimidazolium chloride ionic liquid, [C_4_C_1_im]Cl, and water, using computational NMR spectroscopy. The structure of the complex fluid phase, where the ionic and hydrophobic nature of ionic liquids is further complicated by the addition of water, is first generated by classical Molecular Dynamics (MD) and then validated by calculating the NMR properties with DFT at the ONIOM(B3LYP/cc-pVTZ//B3LYP/3-21G) on clusters extracted during the MD trajectories. Three ionic liquid/water mixtures have been considered with the [C_4_C_1_im]Cl mole fraction of 1.00, 0.50, and 0.01, that is the pure ionic liquid [C_4_C_1_im]Cl, the equimolar [C_4_C_1_im]Cl/water mixture, and a diluted solution of [C_4_C_1_im]Cl in water. A good agreement is obtained with published experimental data that, at the same time, validates the structural features obtained from the MD and the force field used, and provides an example of the power of NMR spectroscopy applied to complex fluid phases.

## 1. Introduction

Computational NMR spectroscopy is a very useful complementary tool for the structural elucidation of organic molecules and natural substances [1,2,3,4]. In cases where the experimental NMR analysis is not capable of revealing with absolute confidence the structure of an unknown compound, a quantum chemical calculation of the NMR properties (normally ^1^H and ^13^C isotropic shielding constants, as well as *J*-couplings) of putative structures can help establish the correct molecular architecture. The computational protocol is based on a two-step sequence: First, a geometry of the proposed structure needs to be produced at some theoretical level, often based on Density Functional Theory (DFT); then the NMR properties are calculated, again often at the DFT-GIAO (Gauge-Including Atomic Orbitals) level, and compared with the available experimental data. The agreement/disagreement, measured by a variety of statistical parameters [5], between experimental and calculated data is an indication of the correctness/incorrectness of the structural proposal. Since normally NMR data are obtained in nonpolar solvents, calculations of a single molecule data, in the gas phase or simply including the long-range solvent reaction field with continuum methods, are sufficiently accurate.

The protocol reliability is rooted on the assumption, nowadays fully validated [6,7], that high level calculations of the NMR properties are extremely accurate, and the NMR spectra of two different molecules cannot be exactly the same just by chance [8], therefore the disagreement between the experiment and calculation can only stem from an erroneous structural arrangement. Conversely, a good match between calculated and experimental data supports the structural proposal. 

In a recent review paper, I have described how such methods of “computational spectroscopy” can be extended to more complex fluid phases, such as Ionic Liquids (ILs) [9]. In such cases, the notion of “structure” of course needs to be revised, since we are no longer dealing with a single molecule but with a bulk phase. If the interest is on the effect of the environment on the chemical shifts of the constituent ions of the IL, namely, the effect of the anions on the cations’ chemical shifts or vice versa, or the effect of a cosolvent in a mixture, then the whole bulk phase and its dynamics have to be considered. One conceptually simple approach, though computationally rather laborious, is to run a Molecular Dynamics (MD) simulation, to extract clusters of appropriate size during the time evolution, and then to calculate the NMR properties of such clusters and simply average the results. This approach has been successfully used in the past for pure ILs with remarkable success [10,11,12]. Again, similarly to the computational protocol used for fully covalent natural substances, the agreement/disagreement of the calculated and experimental data provides an indication of the degree of correctness of the average structure of the bulk phase produced by the MD simulation. 

It is worth mentioning that computational NMR spectroscopy has been also successfully applied to solid-state NMR, for example to study polymorph structures or inorganic systems [13,14,15,16]. In such cases, chemical shift anisotropy information complement the set of NMR parameters that can be used for a comparison with experimental data [17,18,19]. However, the structural elucidation of unknown complex natural substances cannot relinquish the abundance of information, especially concerning proton resonances, available in the high-resolution liquid state NMR spectra.

Mixtures of ILs with water and other solvents are an efficient method to tune the properties of solvents, such as viscosity, solubility, melting point, etc. [20]. It is, therefore, very important to understand the details of the structure of IL/solvents mixtures, in particular when the solvent is water, because of the peculiar structure of ILs, comprising ionic parts as well as alkyl chains that tend to nano-segregate [21,22,23]. To this end, MD simulations are the method of choice, since they reveal the microscopic details of the average structure of ILs mixtures, see Ref. [24] for a recent review. 

**Figure 1:**
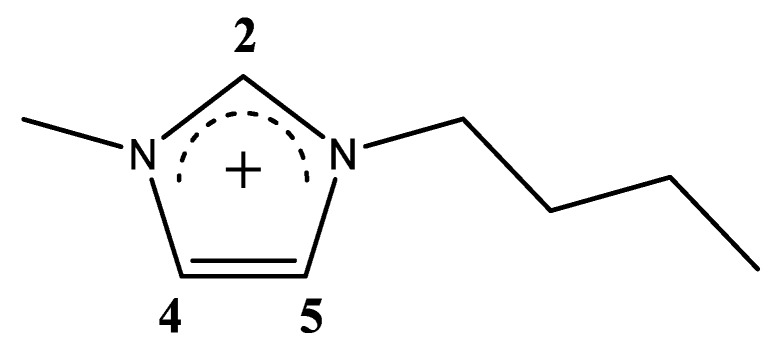
Structural formula of the 1-butyl-3-methylimidazolium cation, [C_4_C_1_im] and atom numbering of the ring positions.

In this work, I present an application of the abovementioned computational protocol to a complex system that is a mixture of 1-butyl-3-methylimidazolium chloride, [C_4_C_1_im]Cl, with water. The structure of the cation is shown in Figure 1. A detailed experimental study, including NMR parameters, have been published in Ref. [25] by Kim and co-workers. The imidazolium proton resonances, especially proton 2, is found to be extremely sensitive to the mixture composition with its shielding constant increasing as the amount of water is increased. A similar trend is observed for protons H4 and H5, while the opposite trend is found for the methyl group of the butyl chain [25].

Thus, first the results of MD simulations of three representative mixtures (a pure IL, an equimolar mixture *x*_IL_ = 0.50, and a diluted system, *x*_IL_ = 0.01) used to provide the average structure, will be discussed. Then, clusters extracted during the trajectories will be used for DFT-NMR calculations. As we will see, the good agreement obtained between the average simulated NMR properties and the experimental NMR data, validates the structural features obtained from the MD simulations.

## 2. Results

### 2.1. Molecular Dynamics Simulations

**Figure 2:**
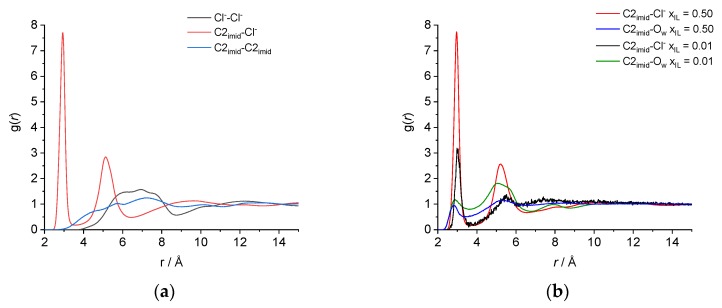
Some representative radial distribution functions (RDF) of (**a**) pure [C_4_C_1_im]Cl and (**b**) [C_4_C_1_im]Cl/water mixtures.

The structure of the three simulated systems can be described by their Radial (RDF) and Spatial (SDF) Distribution Functions. The RDF, or g(*r*), are shown in Figure 2. For the pure IL system, Figure 2a, we note two very strong peaks for the distribution of probability of the distance between the chloride anion and the carbon C2 of the imidazolium ring. The first peak corresponds to the chloride anions hydrogen-bonded to highly acidic hydrogen H2 (its area corresponds to 1.06 anions), while the second peak, larger in width, includes the chloride anions interacting with hydrogens H4 and H5 and surrounding the imidazolium ring at larger distances, having an area corresponding to 4.00 anions. 

Both peaks, however, represent the first shell of solvation of the imidazolium ring, so the value of 6.5 Å, corresponding to the second minimum of the C2_imid_-Cl^−^ RDF is taken as the cutoff radius for the definition of the cluster size, see Computational Methods Section. Consistent with the general structure of ionic liquids, the anion–anion and cation–cation RDF have their first maxima after the first solvation shell. As we can see in Figure 2b, the local structure is not significantly changed in the mixture with a *x*_IL_ = 0.50: The comparison of the chloride—C2 and water oxygen—C2 distance distribution probability reveals that chloride is still strongly interacting with the charged hydrogens of the imidazolium ring, while water has a very weak interaction. It is only for the mixture with a *x*_IL_ = 0.01 that we observe a significant change in the profile of the RDF.

**Figure 3:**
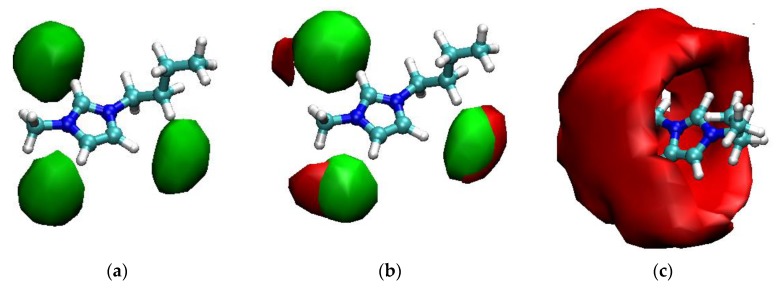
The spatial distribution functions (SDF) of the probability of finding the chloride anion (green) and/or water oxygen (red) around the imidazolium cation for: (**a**) The pure ionic liquid (IL) system, iso-contour value is eleven times higher than in the bulk; (**b**) the [C_4_C_1_im]Cl/water mixture with *x*_IL_ = 0.50, iso-contour value for the chloride (green) is 9.6 times the bulk value, for the water oxygen (red) is seven times the bulk value; (**c**) the [C_4_C_1_im]Cl/water mixture with *x*IL = 0.01, the iso-contour value is for a probability density thirty-two times the bulk value.

A better understanding can be obtained by inspecting the SDF reported in Figure 3. 

Therefore, the water molecules, even when present in a 1:1 ratio, do not significantly disrupt the local structure of the ionic liquid. The mismatch in the probability maxima for oxygen and chloride iso-contours in Figure 3b indicates that water is more likely hydrogen-bonded with the chloride anions rather than replacing them as an acceptor of hydrogen bonds from the acidic hydrogens of the imidazolium ring. Of course, this does not hold anymore when the water is in large excess, now that the imidazolium cation is fully solvated by water, see Figure 3c. However, it appears that water forms a continuum network around the imidazolium ring without having specific interactions and without being strongly localized in front of the acidic hydrogens as it occurs with chlorine.

As described in the Computational Methods Section, from the 10 ns trajectory of each system 100 configurations were selected at regular times separated by 100 ps, and for each configuration a random cation in the box was chosen. Then, a cluster of molecules within a cutoff distance of 6.5 Å from carbon C2 was extracted. The choice of position 2 of the imidazolium ring as the reference is related with the fact that proton 2 NMR is the most sensitive to environmental effects, that is the anion type [26] and mixture composition [25,27]. The clusters were built including any molecule such that at least one atom is within the cutoff radius in order to have integer molecules inside.

**Figure 4:**
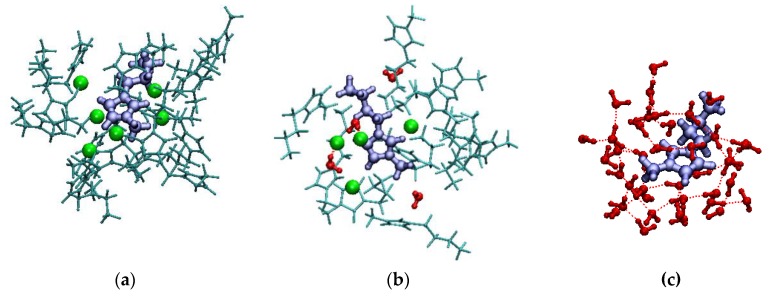
Representative clusters of (**a**) the pure IL system; (**b**) the [C_4_C_1_im]Cl/water mixture with *x*_IL_ = 0.50; (**c**) the [C_4_C_1_im]Cl/water mixture with *x*_IL_ = 0.01. In blue, the thick ball and stick are the central imidazolium cations; in green, the chloride anions; in red, the water molecules; in cyan, the thin ball and stick are the other imidazolium cations.

Three representative clusters are shown in Figure 4.

From Figure 4, it is clearly visible that the discussion on the local structure of the system is confirmed by the visual inspection of the clusters. We note that, by construction, the clusters are expected to be positively charged, since the inclusion of any integral molecule having any atom within the cutoff radius is likely to bring in several imidazolium cations whose, for example, alkyl chain is at less than the cutoff radius from carbon C2. In fact, the average charge of the clusters is +7.8*e* for the pure IL system, +7.0*e* for the [C_4_C_1_im]Cl/water mixture with *x*_IL_ = 0.50, and 1.1*e* for the [C_4_C_1_im]Cl/water mixture with *x*_IL_ = 0.01. These numbers are consistent with the average composition of the clusters reported in Table 1.

The values reported in Table 1 qualitatively agree with the analysis of the structure of the bulk phase of the pure IL and the IL/water mixtures: The addition of an equimolar amount of water does not change significantly the local structure around the imidazolium cation, while the highly diluted system is, of course, very different.

To check if the average structure obtained from the classical MD simulations is indeed correct, the NMR resonances of the imidazolium cation will be calculated following the rationale behind the very well-established computational protocols for structural validation of natural compounds, which has been described in the Introduction. It is expected that subtle variations of the imidazolium chemical shifts with the mixture composition will be a very sensitive probe of the local structure of the systems.

### 2.2. DFT-NMR Calculations

**Figure 5:**
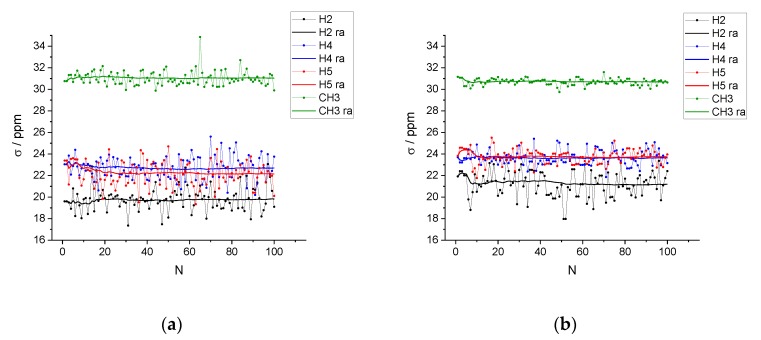
Instantaneous values of the isotropic shielding constant, σ, and running average (ra) for the imidazolium ring protons and the terminal methyl group of the butyl chain. (**a**) Pure IL system; (**b**) [C_4_C_1_im]Cl/water mixture with *x*_IL_ = 0.01.

DFT-NMR calculations were run on the selected clusters using the ONIOM scheme at the levels of theory described in the Computational Methods Section. The instantaneous values of the isotropic shielding constants for the imidazolium ring protons, H2, H4, and H5, and for the terminal methyl group of the butyl chain, are shown in Figure 5 for two systems: The pure IL and the diluted mixture. Qualitatively very similar trends are obtained for the equimolar mixture. 

As mentioned in the Introduction, our study was limited to the NMR resonances experimentally monitored in Ref. [25] with a special attention to proton H2 and its dependence on the mixture composition. As we can see in Figure 5, the instantaneous values are highly fluctuating by several ppm. However, after including 100 configurations, the average value converges within an estimated error, for all cases, of about 0.1 ppm or less. This precision is sufficient for a semiquantitative analysis and comparison with experimental values. The calculated average isotropic shielding constants are also reported in Table 2.

As we can see, starting from the pure IL system, the three imidazolium proton resonances are increasingly shielded (that corresponds to a decreasing chemical shift in the ^1^H NMR spectrum) as the composition of the mixture varies by the addition of water. In contrast, the resonance of the CH_3_ group of the butyl chain is little affected for the composition between *x*_IL_ = 1.0–0.5 while it is de-shielded in the highly diluted mixture with *x*_IL_ = 0.01. 

**Figure 6:**
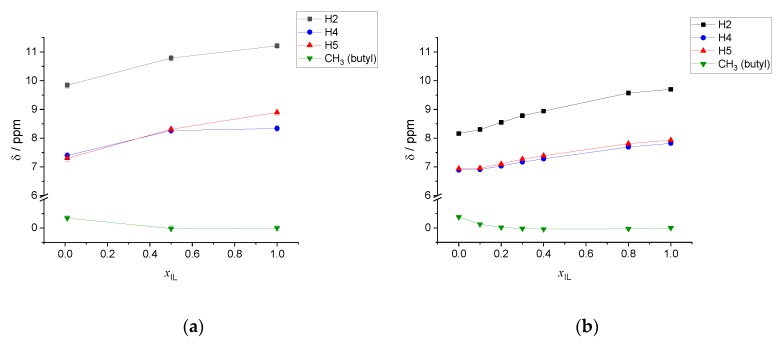
Chemical shifts as a function of the composition of the [C_4_C_1_im]Cl/water mixture. (**a**) Average calculated values. (**b**) Experimental data from Ref. [25]. Connecting lines are merely a guide to the eyes.

In Figure 6, we show the dependence of the chemical shifts as a function of the mixture composition. For both the experimental [25] and calculated values, the reference has been taken as the chemical shift in the pure IL system of the methyl group of the butyl chain, which is, therefore, arbitrarily set to 0 ppm. This allows us to bypass the calculation of the shielding constant of the experimental NMR reference, tetramethylsylane, that should be conducted at the same level of theory (classical MD simulation followed by cluster extraction). The agreement between calculated and experimental data is rather good, though not completely quantitative. First, all trends are correctly reproduced: Decreasing the amount of water leads to a significant de-shielding of the ring resonances (1–2 ppm) while the butyl resonance is shielded by ca. 0.5 ppm. Moreover, the very similar H4 and H5 ring proton resonances are qualitatively calculated to be very close, except for the pure IL system where, though the resonances are in the correct order, the calculated difference is appreciably larger than in the experiments. Furthermore, the calculated chemical shift of H2 is consistently higher (more de-shielded) than the experimental value. 

**Figure 7:**
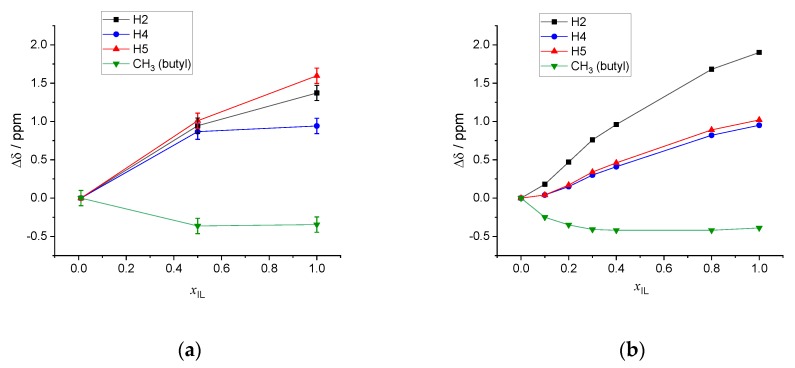
Relative chemical shift variation as a function of the composition of the [C_4_C_1_im]Cl/water mixture. (**a**) Average calculated values. (**b**) Experimental data from Ref. [25]. Connecting lines are merely a guide to the eyes.

It is also interesting to report the relative variations, taking as a reference the chemical shift in the high dilution limit for each proton and defining the relative shift for any composition *x*_IL_ as Δδ(*x*_IL_) = σ(*x*_IL_) − σ(0.01) for both the results of the calculations and the experiments of Ref. [25]. The final averaged results of the DFT-NMR calculations are reported in Figure 7a, while in Figure 7b, the analogous experimental values obtained from Ref. [25] are shown. 

As discussed already concerning Figure 6, the results of the calculations are almost in quantitative agreement with the experiments, with the only exception of the result of H5 in the pure IL, which appears slightly more de-shielded than the experimental value. It is difficult to pinpoint a specific reason for the slightly poorer performance of the protocol for H5 in the pure IL phase. In Ref. [11], it was shown that even very minor changes in the distribution of chloride anions around 1-ethyl-3-methylimidazolium chloride may have significant effects on the final calculated proton chemical shift. With this exception, however, the remaining trends are very nicely reproduced by the calculations. 

## 3. Discussion

Considering the notable sensitivity of the NMR parameters to structural features, it can be stated that the overall agreement between experimental and calculated trends is indeed rather good. Therefore, we can retrospectively investigate what are the features of the average structure of the ILs/water mixtures that are responsible for the observed shielding trends. First, it is necessary to stress that the MD simulations were run with constrained C–H bond lengths, in order to be able to use a time step of 1 fs. The bond lengths for the selected force field are 1.08 Å (C–H of the imidazolium ring) and 1.09 Å (C–H of the alkyl groups). For DFT-NMR calculations of organic compounds and natural substances, it is normally required to have accurate geometries as a starting point [28]. However, in our system, the errors introduced by the noncomplete treatment of the dynamics and the complexity of the bulk phase structure, not to mention the classical and nonpolarizable nature of the force field, are larger than the errors coming from a geometry not optimized at a high DFT level of theory. 

The second important point is that NMR properties are indeed “local” [29,30], meaning that they are not influenced by long-range structural features of the system. Environmental effects, such as the mixture composition, indeed are significant, but the inclusion of the first solvation shell centered on the reference imidazolium ring appears to be sufficient in order to obtain a semiquantitative agreement with the experimental data. Moreover, in the NMR-GIAO calculation, once the reference molecule is described at a high level of theory, the treatment of the solvation shell can be run at a very low QM level without significantly compromising the final outcome. 

Finally, let us see how the ionic liquid structure changes with the addition of water. For the pure IL system, the anion is found to be preferentially located, on average, in the plane of the imidazolium ring. The strong interaction between the anion and the acidic ring protons is responsible for the high de-shielding of the resonances. The addition of water, as found from the MD simulation and confirmed by comparison of the calculated NMR data with experiments, does not significantly alter the local structure of the imidazolium cation: Due to the small size of water, even in an equimolar mixture, the acidic ring hydrogens are preferentially interacting with the anion, while water is more likely to be found hydrogen-bonded to the chloride anion as a hydrogen-bond donor, rather than with the ring hydrogens as a hydrogen-bond acceptor. 

## 4. Computational Methods

Classical Molecular Dynamics (MD) simulations were run with the software package Gromacs v. 2019.2 [31,32]. I have used the nonpolarizable force field of Ref. [33] for the imidazolium [C_4_C_1_im] cation, the parameters of the CL&AP force field for the chloride anion [34], and the TIP3P water model [35]. Three systems were prepared, a pure IL system composed of 400 [C_4_C_1_im]Cl ion pairs; a water mixture system with an IL mole fraction *x*_IL_ = 0.50, composed of 200 [C_4_C_1_im]Cl ion pairs and 200 water molecules; and a water mixture system composed of 10 [C_4_C_1_im]Cl ion pairs and 1000 water molecules, therefore with an IL mole fraction of *x*_IL_ = 0.0099 (approximated to 0.01 hereafter). Initial boxes were prepared with the software package *packmol* in a low density box [36], energy minimized and first heated at 800 K for 1 ns at a constant volume. Then, the temperature was decreased in steps of 100 K down to 300 K, each run still for 1 ns. The boxes were then equilibrated at 300 K for an additional 1 ns in the isothermal-isobaric (NPT) ensemble before the production run of 10 ns, still at a constant pressure (1 atm) and temperature (300 K). Equilibrium densities for the three systems are shown in Table 3 and are in good agreement with the available experimental data.

C–H bonds were constrained by the LINCS algorithm [39]. The leap-frog integrator was used with a time step of 1 fs and a cutoff of 10 Å for the van der Waals and short-range electrostatic interaction. The Particle-Mesh-Ewald (PME) [40] technique was used to handle the long-range electrostatic interaction with an interpolation order of 4. Simulations were run in the NPT ensemble using the Berendsen thermostat and barostat [41] with applied isotropic periodic boundary conditions. Configurations were saved every 1 ps for further analysis.

From the trajectory files obtained during the 10 ns production run of each system, 100 frames were selected, one every 100 ps, and from each frame a cluster centered on the C2 carbon of a randomly selected [C_4_C_1_im] cation was extracted. The cluster contains the selected cation plus all molecules within a cutoff radius of 6.5 Å in order to make sure to include at least the first shell of counter-anion, see the discussion of the radial distribution functions in the Results Section. Any molecule having at least one atom within the cutoff radius was included in the cluster. The clusters were used without further optimization for the subsequent DFT-NMR calculations. The size of the clusters varied from a minimum of 230 atoms, to a maximum of 405 for the pure IL system; from a minimum of 248 to a maximum of 404 for the system at *x*_IL_ = 0.50; and from a minimum of 109 to a maximum of 190 atoms for the system at *x*_IL_ = 0.01. Spatial distribution functions are produced with the software *travis* [42] and visualized with the software *VMD* [43].

DFT-NMR calculations were run using the ONIOM [44] scheme for NMR-GIAO calculations implemented in Gaussian 16 [45]. The cluster extracted from the trajectory is divided into a high layer, that is the central [C_4_C_1_im] cation, described at the high level B3LYP/cc-pVTZ, and a low layer, all the remaining atoms, described at the low level B3LYP/3-21G. The large size of the cluster does not allow using larger basis sets for the low layer, this is however, sufficient to represent the charge distribution and the effects of the polarization on the central cation. Layers clearly differ in the total charge, while in all cases the systems are singlets. The isotropic NMR-GIAO shielding constant, σ, within the ONIOM scheme, is then obtained as σ = σ(HL, model) − σ(LL, model) + σ(LL, real), where HL and LL means High Level and Low Level, respectively, while the labels model and real indicate the central [C_4_C_1_im] cation and the entire cluster, respectively. 

## 5. Conclusions

In this work, I have presented a computational NMR investigation of the binary mixture structure of 1-butyl-3-methylimidazolium chloride with water. A microscopic description of the average bulk structure of the system has been obtained from classical MD simulations. In order to validate the structure and, therefore, the force field, the average shielding constants of the imidazolium ring protons and the methyl group of the butyl chain (which show a characteristic trend as a function of the mixture composition) have been calculated and compared with NMR experimental data reported in the literature [25]. The good agreement observed confirms the goodness of the classical force field used and the structural features reported. Moreover, it provides an example of the power and scope of the computational spectroscopy for the investigation of bulk phases. 

## Figures and Tables

**Table 1:** Average values of the number of cations, <N_CAT_>, number of anions, <N_AN_>, number of water molecules, <N_W_>, and charge <q> of the clusters obtained for each system.

x_IL_	<N_CAT_>	<N_AN_>	<N_W_>	<q>
1.00	12.9	5.1	0	+7.8
0.50	11.9	4.9	5.6	+7.0
0.01	1.3	0.2	38.8	+1.1

**Table 2:** Calculated average isotropic absolute shielding constants, σ, and corresponding chemical shifts, δ, in parenthesis calculated with respect to the butyl CH_3_ resonance, in ppm. Error on the mean, after considering 100 configurations, is ±0.1 ppm.

x_IL_	σ (δ) H2	σ (δ) H4	σ (δ) H5	σ (δ) CH3 Butyl
1.00	19.82 (11.21)	22.70 (8.34)	22.14 (8.90)	31.04 (0.00)
0.50	20.25 (10.79)	22.77 (8.26)	22.72 (8.31)	31.05 (−0.02)
0.01	21.19 (9.84)	23.64 7.40	23.73 (7.30)	30.69 (0.35)

**Table 3:** Some properties of the simulated systems in the NPT ensemble (300 K, 1 atm): Equilibrium average density, <d>; average length of the cubic box side, L; total number of atoms in the box, N; composition, N_IP_ ion pairs + N_W_ water molecules.

x_IL_	<d> (g/mL)	<L> (Å)	N Atoms	N_IP_ + N_W_
1.00	1.11 ^a^	47.04	10,025	400 + 0
0.50	1.15 ^b^	38.16	5800	200 + 200
0.01	1.01 ^c^	31.96	3260	10 + 1000

^a^ Experimental density is 1.07941 g/mL at 303.15 K, from Ref. [37]. ^b^ Experimental density is 1.07853 g/mL for *x*_IL_ = 0.4083 at 303.15 K, from Ref. [38]. ^c^ Experimental density is 1.0038 g/mL for *x*_IL_ = 0.0121 at 303.15 K, from Ref. [38].
